# Concurrent Myotonic Dystrophy and Inflammatory Myopathy in a Patient with HIV/AIDS

**DOI:** 10.1155/2021/9998415

**Published:** 2021-05-20

**Authors:** Akash Gupta, Anita Huttner, Marwan M. Azar

**Affiliations:** ^1^Department of Medicine, Yale School of Medicine, New Haven, CT 06520, USA; ^2^Department of Pathology, Yale School of Medicine, New Haven, CT 06520, USA; ^3^Section of Infectious Diseases, Department of Medicine, Yale School of Medicine, New Haven, CT 06520, USA

## Abstract

Neuromuscular complications are common in patients with HIV/AIDS at any stage of the disease process. Myopathies can be secondary to antiretroviral therapy, HIV myositis itself, or other etiologies. Here, we present the case of a middle-aged male with HIV who presented with myalgias and was diagnosed with myotonic dystrophy and HIV-associated polymyositis after extensive workup including clinical history and physical exam, laboratory markers, electromyogram, and muscle biopsy. This case illustrates the importance of a comprehensive workup for myopathy in HIV/AIDS and the possibility of multiple concurrent conditions.

## 1. Introduction

Neuromuscular complications are common among patients with HIV infection and may occur at any stage of the disease process. HIV-related myopathies may result from an HIV-related myositis, mitochondrial toxicities secondary to antiretroviral therapy (ART), especially nucleoside reverse transcriptase inhibitors, or both [1]. Secondary myopathies, due to infectious, neoplastic, metabolic, or autoimmune processes, may also occur among patients with HIV but are rare [1–4].

We report the case of a 48-year-old HIV-positive male who presented with myalgias and was found to have a HIV-associated inflammatory myopathy with concurrent inherited myotonic dystrophy. Very few reports of myotonic dystrophy in HIV-positive patients have been described; a broad workup may be needed to determine the etiology of myopathy in HIV-infected patients.

## 2. Case Report

Upon presentation, the patient reported three weeks of diffuse muscle aches with stiffness in his lower extremities. His medical history was also notable for depression with psychotic features, treated with risperidone as well as cocaine use, and HIV/AIDS (prior CD4 nadir was 419 cells/mm^3^), diagnosed 20 years ago. He had been off ART for the past three years (last regimen was tenofovir/emtricitabine, atazanavir, and ritonavir) and lost to follow up. Physical exam was notable for hypertrophied biceps and calf muscles. Labs demonstrated a CD4 count of 185 cells/mm^3^, a HIV viral load of 54,900 copies/mL, and elevated creatine kinase (CK) of 1667 U/L with normal kidney function. The cause of his muscle weakness was attributed to rhabdomyolysis from recent adjustment of his risperidone dosing and active cocaine use. Risperidone was stopped, and he was given diphenhydramine and benztropine with some improvement in his symptoms. Further infectious workup, including blood cultures, hepatitis B and C serologies, and QuantiFERON-TB gold, was negative. The patient was left against medical advice prior to ART reinitiation.

**Figure 1:**
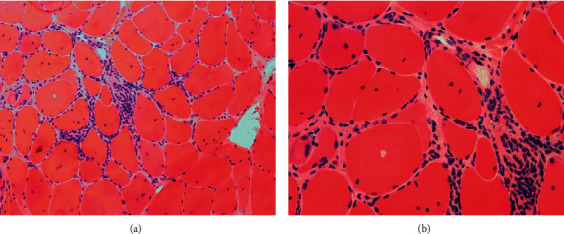
Histopathologic images of the muscle biopsy. Hematoxylin and eosin staining ((a), 10x power) is remarkable for the presence of numerous endomysial inflammatory cells, which lead to myophagocytosis, myofiber degeneration, and atrophy. (b) A higher power image (20x) and demonstrates numerous internalized nuclei which involve the majority of myofibers, and one myofiber with an intramyofibrillar vacuole. The inflammatory cells are predominately composed of CD3 and CD8 positive T-lymphocytes and CD68 positive macrophages (not shown).

He returned to the hospital 1 week later with worsening muscle pain, particularly in his calves and forearms. At this point, a broad workup for primary and secondary myopathies was performed. ANA, anti-Jo-1, and myasthenia gravis antibodies were negative; thyroid and cortisol levels were normal. An electromyogram (EMG) showed myotonic discharges and was consistent with a myopathy and a disorder of muscle membrane instability. A muscle biopsy of the left calf was performed, and pathology revealed central nuclear migration within the majority of myofibers and a significant inflammatory infiltrate, highly suggestive of myotonic dystrophy and HIV myopathy (Figures 1(a) and 1(b)). He later revealed similar muscular complaints in other family members on the maternal side. Confirmatory genetic testing was planned in the outpatient setting, but the patient was unfortunately lost to follow up after discharge.

## 3. Discussion

HIV-associated myopathy, also known as HIV-associated polymyositis (“HIV-PM”), is the most common muscular disorder associated with HIV infection, but the prevalence remains unknown [1–4]. In a retrospective review by Johnson et al. [5], 64 patients attending an HIV outpatient clinic were referred to a rheumatology clinic for muscle complaints or elevated CK; of this group, only 13 demonstrated HIV-PM.

HIV-PM is clinically and pathologically similar to autoimmune polymyositis in HIV-negative individuals [1–3, 6]. On histopathology, HIV-associated inflammatory myopathy manifests as an inflammatory infiltrate within the muscle tissue, predominantly comprised of CD8+ T lymphocytes and macrophages. In a series of 15 patients with HIV-PM, histologic features were identical to those seen in polymyositis in non-HIV affected individuals [7]. In our patient, the diagnosis of HIV-PM was made, in accordance with the 2017 European League Against Rheumatism/American College of Rheumatology (EULAR/ACR) criteria for polymyositis, based on absence of skin manifestations, the presence of muscle weakness in his legs, and findings on muscle biopsy [8].

The pathogenesis of HIV-PM remains largely unknown, as the direct role of HIV has not been well defined [1, 5–7, 9]. One hypothesis is that inflammatory cell-derived cytokines or lymphokines lead to muscle fiber damage, precipitating muscle antigen exposure and a process where muscle fibers themselves act as antigen presenting cells, triggering an autoimmune response [9]. Notably, the severity of inflammatory infiltrate has not been shown to correlate with a patient's symptoms or stage of HIV infection [1, 5–7, 9].

The presence of significant fiber variability and internalized nuclei in majority of the myofibers in our patient, coupled with the patient's family history and EMG findings, strongly suggested a concurrent myotonic dystrophy. Genetic testing is typically confirmatory for the diagnosis of myotonic dystrophy; however, in instances where it is unavailable like in our patient, the diagnosis can be made using clinical history and exam, EMG, and muscle biopsy findings [10]. The histopathology findings of myotonic dystrophy are often sufficient for the diagnosis in the appropriate clinical context [11–13].

The new diagnosis of an inherited myopathy was especially notable, given the patient's age. To our knowledge, there is only one other report describing a patient with HIV/AIDS and myotonic dystrophy who developed pilomatricomas two years after the AIDS diagnosis [14].

This case illustrates the broad differential diagnosis of myopathy in a patient with HIV/AIDS, the possibility of missed diagnoses of genetic diseases in adult patients, the occurrence of multiple concurrent conditions at once, and the need for a comprehensive workup including a muscle biopsy to clinch the diagnoses.

## Data Availability

The patient data used for this report are restricted by the Yale Institutional Review Board to protect patient privacy. Data are available for only authors who meet the criteria for access to confidential data.
